# Comprehensive Simulation Framework for Space–Air–Ground Integrated Network Propagation Channel Research

**DOI:** 10.3390/s23229207

**Published:** 2023-11-16

**Authors:** Zekai Zhang, Shaoyang Song, Jingzehua Xu, Ziyuan Wang, Xiangwang Hou, Ming Zeng, Wei Men, Yong Ren

**Affiliations:** 1 Tsinghua Shenzhen International Graduate School, Tsinghua University, Shenzhen 518055, China; zhangzej21@mails.tsinghua.edu.cn (Z.Z.); xjzh23@mails.tsinghua.edu.cn (J.X.); 2 Yangtze Delta Region Academy of Beijing Institute of Technology, Jiaxing 314000, China; 1120191930@bit.edu.cn; 3 School of Information and Electronics, Beijing Institute of Technology, Beijing 100081, China; mzengzm@163.com; 4 Department of Electronic Engineering, Tsinghua University, Beijing 100084, China; wangziyu21@mails.tsinghua.edu.cn (Z.W.); hxw21@mails.tsinghua.edu.cn (X.H.); reny@tsinghua.edu.cn (Y.R.)

**Keywords:** space–air–ground integrated network, propagation channel research, scenario identification, QuaDRiGa, satellite channel simulation platform

## Abstract

The space–air–ground integrated network (SAGIN) represents a pivotal component within the realm of next-generation mobile communication technologies, owing to its established reliability and adaptable coverage capabilities. Central to the advancement of SAGIN is propagation channel research due to its critical role in aiding network system design and resource deployment. Nevertheless, real-world propagation channel research faces challenges in data collection, deployment, and testing. Consequently, this paper designs a comprehensive simulation framework tailored to facilitate SAGIN propagation channel research. The framework integrates the open source QuaDRiGa platform and the self-developed satellite channel simulation platform to simulate communication channels across diverse scenarios, and also integrates data processing, intelligent identification, algorithm optimization modules in a modular way to process the simulated data. We also provide a case study of scenario identification, in which typical channel features are extracted based on channel impulse response (CIR) data, and recognition models based on different artificial intelligence algorithms are constructed and compared.

## 1. Introduction

The 6G networks are envisioned to be highly reliable, with wide coverage and low latency, enabling a wide range of promising services and applications [1,2,3]. As 6G envisions an amalgamation of communication, computation, and sensing, it calls for a communication infrastructure that surpasses existing boundaries. SAGIN, by seamlessly integrating space, air, and ground communication domains, offers a fertile ground for the manifestation of 6G’s transformative attributes [4,5]. The network’s capacity for high data rates, ultra-low latency, massive connectivity, and reliable communication aligns harmoniously with 6G’s overarching objectives [1]. Unfortunately, due to constraints such as limited resources and complex structure, it is difficult for SAGIN to achieve optimal performance. Therefore, it is necessary to carry out research on SAGIN network design, optimization, and control, and the study on propagation channels is the basis of these topics [6].

The heterogeneous nature of SAGIN necessitates a nuanced comprehension of communication propagation channels that spans diverse domains. In space, air, and ground, scenarios encompassing line-of-sight (LOS) and non-line-of-sight (NLOS) propagation, multipath effects, and atmospheric conditions influence communication efficacy and reliability [7]. Profound insights into these communication propagation channels are pivotal to developing communication strategies for SAGIN that optimize performance and reliability. The research of communication propagation channels can be roughly divided into channel modeling and scenario identification. For channel modeling, it aims to reveal the relationship between physical environment and channel attributes, that is, to characterize wireless channels through key channel parameters, and to find the correlation between different parameters and environments for modeling. Then, the established channel model can be used to predict other time-domain, frequency-domain, and spatial channel characteristics that have not yet been measured. Environmental changes will affect the design of channel model, physical layer, and network layer, especially for intelligent transportation systems such as vehicle networks [8,9,10]. Real-time scenario identification is conducive to the adaptive adjustment of complex network architectures such as SAGIN, that is, appropriate channel model and transmission mode can be selected for specific environments to improve communication quality.

The above analysis shows the significance of studying SAGIN communication propagation channels, which can feed back to the upper layer design and adaptive adjustment of SAGIN, and further promote the development of 6G mobile network. However, communication propagation channel research is faced with many challenges, one of which is the lack of intelligent and efficient analysis methods [6]. Traditional research methods based on statistics are easily restricted by experimental conditions, and it is difficult to obtain sufficient measurement data, and the process is time-consuming and laborious [11]. In response to this challenge, more and more researchers have begun to learn from machine learning (ML)- and artificial intelligence (AI)-related technologies to study channel feature extraction, channel estimation, and wireless communication scenario recognition [12,13,14]. In addition, it is often necessary to collect a large amount of data to support propagation channel research. However, the data collected in the actual environment is incomplete and noisy, and the measurement process is time-consuming and labor-intensive. Therefore, adopting simulation platform to simulate channel data has become a new way [15]. At present, the relatively mature wireless channel simulation platform is QuaDRiGa [16], but it is not suitable for space domain simulation, so it is difficult to use it in the research of communication propagation channels of heterogeneous networks such as SAGIN.

Enlightened by the previous analysis, to further understand the SAGIN propagation channel, this paper designs a comprehensive simulation framework, which can simulate the communication channel data across different scenarios and support users to process and analyze the obtained channel data. The primary contributions of this article are summarized as follows:

- We design a comprehensive simulation framework for data simulation, processing, and analysis of SAGIN communication propagation channels, which provides a paradigm for researchers.
- To make up for the shortcomings of existing channel simulation platforms in space domain simulation, the framework integrates a self-developed modular low-orbit satellite communication channel simulation platform.
- Based on integrated simulation platforms, we first simulate different communication scenarios, and then construct scenario recognition cases, in which the importance of different channel characteristics for different link types is analyzed, and the recognition effects of different artificial intelligence algorithms are analyzed.

The rest of this article is organized as follows. Related work is stated in Section 2. In Section 3, we introduce the proposed simulation framework and self-developed low-orbit satellite channel simulation platform. In Section 4, the channel characteristics and algorithms used in this paper are introduced. In Section 5, we show the generation of simulation data, extract channel features based on the simulation data, and evaluate the importance of different features to different scenario classifications, and finally study the scenario recognition cases. Finally, the conclusions and future work are given in Section 6.

## 2. Related Work

This section introduces the state-of-the-art research in the field of SAGIN propagation channels and analyzes the advantages and disadvantages of different solutions.

The traditional statistical analysis method needs a lot of expert annotation work and cannot meet the demand of time-varying transmission channel research. Inspired by the power of machine learning and artificial intelligence, researchers have begun to use them to carry out comprehensive research on channel characterization/modeling and prediction [11]. Yang et al. [17] used K-means to carry out scenario recognition based on multi-dimensional channel features. Based on real-time measurement data, AlHajri et al. [18] used weighted KNN to realize classification and recognition of different indoor environments. Oroza et al. analyzed the performance of random forest, AdaBoost, and KNN in estimating the received signal intensity [19]. Based on initial parameters such as propagation distance, link elevation angle, and visibility conditions, Zhang et al. [20] established a prediction model of UAV channels by using random forest and KNN method. In addition, artificial neural networks have shown great potential in the research of communication transmission channels. In [21,22], backpropagation (BP) artificial neural networks and convolutional neural networks (CNNs) were adopted and trained with channel statistics to recognize LoS/NLoS scenarios, respectively. In order to avoid the gradient disappearance problem, a single-layer MLP network was proposed to obtain the path loss of a specific band ultra-wideband (UWB) channel [23]. The communication scenarios studied in the above literatures are all ground or low-altitude networks, while the studies [24,25], respectively, used generalized artificial neural networks and long short-term memory networks in the prediction of satellite communication channels, and predicted the received power through weather information such as temperature and humidity and relative speed.

The development of simulation platform plays an important role in evaluating the communication performance of SAGIN [15]. On the one hand, the design of the training database is the key to any ML- or AI-based schemes, and it is time-consuming and difficult to expect to obtain a large amount of data through actual measurements. In this case, using the simulation platform to synthesize data to build the training database is an effective method. On the other hand, any algorithms used for SAGIN evaluation need to be continuously optimized to improve performance, and it is also very challenging to deploy these algorithms for experiments in practice, in which case, simulators can be used instead of unnecessary practical experiments for pre-training. At present, a lot of work has been performed to evaluate the performance of ground networks using network simulation platforms such as OPNET [26], Network Simulator NS.3 [27], and MATLAB/Simulink [28]. Researchers combined VISSIM, NS-2, etc., to evaluate the communication coverage capability of the air networks; the transmission delay and data offloading problems of satellite networks were studied based on these simulation tools [29,30]. However, most of these works focus on a single network segment of ground, air, and space, and there are no effective suggestions for a unified simulation of SAG environments. 

The key motivation of this paper is to develop a framework for SAGIN propagation channel research to provide an example for researchers to guide how to simulate channel data in SAG environment by integrating different simulation tools, how to extract features from the obtained data to make training sets, and how to use intelligent algorithms to build models to carry out scenario recognition and other related research.

## 3. Framework Design and Platform Implementation

In this section, the comprehensive simulation framework for SAGIN propagation channel research is designed first, and then the modular low-orbit satellite communication channel simulation platform is introduced to simulate space domain. Finally, the definitions of commonly used channel features are given for subsequent data processing.

### 3.1. Overall Introduction

**Figure 1:**
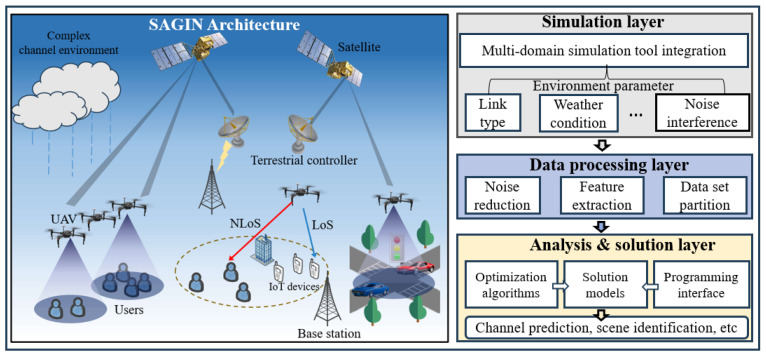
Illustration of simulation framework for SAGIN propagation channel research.

The left half of Figure 1 shows the SAGIN channel propagation scenario considered in this paper. This propagation scenario mainly consists of a space network including low-earth orbit satellites, aerial network including aerial base stations represented by UAVs, and ground network including IoT devices, mobile users, vehicle users, and ground stations that communicate with satellites [31]. This propagation scenario includes a variety of link types such as space-to-space, space-to-air, space-to-ground, air-to-air, air-to-ground, and ground-to-ground, and these channels can be divided into LoS and NloS types. At the same time, these channels are also subject to the interference from complex environments such as rainfall and noise. To study the complex propagation environments, the SAGIN propagation channel comprehensive simulation framework, shown in the right half of Figure 1, is intended to provide a standard example for researchers. It can simulate channels in different communication scenarios of SAGIN and generate simulation data for subsequent model training and testing. The framework consists of three layers of simulation layer, data processing layer, analysis and solution layer; each layer has specified functions and supports the previous layer. In addition, due to the reserved programming interface, the framework supports secondary development. The main components of the framework are described in detail as follows:

- Simulation layer: Existing channel simulation platforms and self-developed platforms are integrated in this layer to simulate channels and generate channel data of different link types. In addition to link types, environmental variables such as weather conditions and noise can also be considered. In this paper, the QuaDRiGa channel simulation platform was used to generate channel data for wireless communication scenarios with link types including ground-to-ground, air-to-ground, and air-to-air. Due to the relatively high speed of low-orbit satellites, the QuaDRiGa platform is not suitable for simulation. Therefore, we develop a low-orbit satellite communication channel simulation platform to generate channel data for wireless communication scenarios with link types of space-to-air, space-to-ground, and space-to-space.
- Data processing layer: This layer is responsible for processing the data generated by the simulation layer, including de-noising, feature extraction, and partitioning the data set for the subsequent training and testing of the model.
- Analysis and solution layer: Machine learning and artificial intelligence technologies have achieved excellent performance in the field of channel research, such as channel prediction and scenario recognition, but the selection of methods and parameters will affect the accuracy of the model. Therefore, this layer integrates some commonly used solving models and is equipped with optimization algorithms. Users can also verify their algorithm models through programming interfaces such as Matlab and Python.

### 3.2. Modular Low Orbit Satellite Communication Channel Simulation Platform

**Figure 2:**
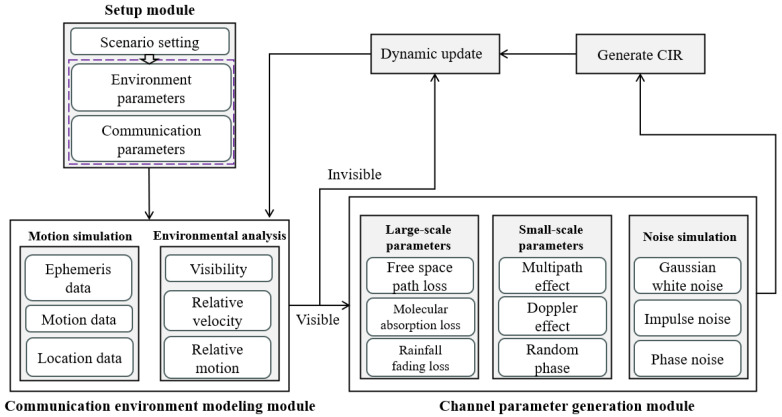
Communication channel modeling flow chart of modular low-orbit satellite simulation platform [32].

To make up for the shortcomings of the QuaDRiGa platform in space domain simulation, we developed a modular low-orbit satellite communication channel simulation platform which can realize channel modeling and simulation; it can generate large- or small-scale parameters and channel impulse responses corresponding to satellite communication channels according to set parameters, which has certain applicability to different scenarios. The platform can change the communication scenario by setting relevant parameters, such as changing the distance between the satellite and the ground to adjust the link type; setting rainfall rate to adjust the weather conditions; setting the signal-to-noise ratio to adjust the noise situation, etc. The modular low-orbit satellite communication channel model is mainly composed of a setting module, communication environment modeling module, channel parameter generation module, and dynamic update module. The channel modeling process is shown in Figure 2. 

As shown in Figure 2, before generating channel data, the communication scenario is first set, including environment parameter settings and communication parameter settings. According to the set parameters, the communication scenario of the satellite is modeled, and ephemeris data, position data, and motion data are obtained immediately so as to conduct real-time dynamic simulation, and analyze the visibility, relative speed, and relative motion of the receiver and the sender. If the channel between the receiving and sending terminals of satellite communication is the LoS channel, the program automatically generates large-scale and small-scale parameters, including free space path loss, molecular absorption loss, rainfall fading loss, multipath effect, Doppler effect, random phase, etc., and the channel impulse response is obtained by the superposition of Gaussian white noise, pulse noise, and phase noise. If the channel between the receiving and sending terminals of satellite communication is the NLoS channel, the channel impulse response is generated and represented by the corresponding needle diagram. Finally, the time state is updated to realize real-time dynamic simulation. In order to extract the channel characteristics of the channel data, all the data in this paper are intercepted from the real-time dynamic simulation data at a certain time to approximate the collection of static channel data.

### 3.3. Channel Feature Selection

The channel data obtained from the simulation layer need to be pre-processed, such as invalid multipath culling and LoS/NloS calibration, and then stored in the database. Before using these original multipath channel data to identify the scenarios, it is necessary to obtain key channel characteristics such as path loss, shadow fading, Rice *K* factor, and Doppler shift by mathematical statistics. Then, the most relevant features are selected for model training through importance analysis. The important notations are listed in Table 1, and the definitions of common channel characteristics are given below [33].

(1) ***Path loss:*** It describes the gradual attenuation of a signal as it propagates over a distance, typically in wireless communication scenarios, which is given by Formula (1):

(1) $$PL=B+10\alpha {log}_{10}\left(\frac{l}{l_{0}}\right)+p\;\;\;\left(l\geq l_{0}\right),$$

where α is the path loss factor corresponding to the specific scenario, *B* is the decibel path loss at distance $l_{0}$, l is the distance between transmitter and receiver, and p is the shadow fading.

(2) ***Shadowing:*** It refers to the phenomenon where electromagnetic waves encounter obstacles, such as buildings, along their propagation path, resulting in the creation of shadow regions that impact signal strength. In practical transmission scenarios, the widely employed model for describing shadowing effects is the lognormal shadow model:

(2) $$P\left(\psi \right)=\frac{\xi }{\sqrt{2\pi }\sigma_{\psi_{dB}}\psi }exp\left[-\frac{{\left(10{log}_{10}\psi -\mu_{\psi dB}\right)}^{2}}{2\sigma_{\psi_{dB}}^{2}}\right]\;\;\left(\psi >0\right),$$

where ξ=10/ln10, $\mu_{\psi dB}$ is the mean of $\psi_{dB},$ $\psi_{dB}=10{log}_{10}\psi$, and $\sigma_{\psi_{dB}}$ is the standard deviation of $\psi_{dB}$.

(3) ***K-factor:*** The Rice fading channel quality factor (*K*-factor) is defined as the ratio of the LoS path signal power to the power of multipath fading within the channel. This factor represents the complexity of signal propagation; a lower *K* factor indicates that the signal power of the LoS path in the signal propagation path is higher and the channel quality is better, and vice versa.

(3) $$K\left(dB\right)=10lg\frac{P_{los}}{p_{nlos}},$$

where $P_{los}$ is the LoS path signal power and $p_{nlos}$ is the multipath fading power.

(4) ***Average time delay:*** Defined as the first moment of the delay power spectrum:

(4) $$\bar{\tau }=\frac{\sum_{k}a_{k}^{2}\tau_{k}}{\sum_{k}a_{k}^{2}}=\frac{\sum_{k}P(\tau_{k})\tau_{k}}{\sum_{k}P(\tau_{k})},$$

where *k* is the number of paths at the current time, $P(\tau_{k})$ is the energy of the *k*-th path, $a_{k}$ is the amplitude, and $\tau_{k}$ is the delay of the *k*-th path.

(5) ***Doppler shift:*** When the mobile station moves towards the base station, the frequency of the electromagnetic wave signal received by the mobile station will become higher. When the mobile station is far away from the base station, the signal frequency received by the mobile station will become lower, resulting in a Doppler shift between the received signal frequency and the transmitted signal frequency.

(5) $$f_{d}=\frac{v}{\lambda }cos\theta ,$$

where θ is the angle of reach, v is the receiver moving speed, and λ is the wavelength.

## 4. Algorithms for Scenario Identification

Many machine learning and artificial intelligence algorithms can be used for propagation channel research. In order to evaluate the performance of different algorithms, the following representative algorithms are considered in this paper for the construction of scenario recognition models [18].

(1) ***Decision Trees:*** Decision trees build a tree-like model by dividing the data set into smaller subsets and splitting them based on feature attributes such as information gain, gain ratio, and Gini index. Each internal node represents a criterion for a feature attribute, and each leaf node represents a category or output result. By following the path from the root node to the leaf node, the class or output of the sample can be predicted based on the values of the feature attributes. The information gain of attribute b is defined as follows:

(6) $$Ga\left(E,b\right)=En\left(E\right)-\sum_{v=1}^{V}\frac{\left|E^{v}\right|}{\left|E\right|}En\left(E^{v}\right),$$

(7) $$En\left(E\right)=-\sum_{i=1}^{\left|o\right|}p_{i}{log}_{2}p_{i},$$

where E is the current sample set, i is the class of samples, o is the total class number, $p_{i}$ is the proportion of class i samples, v is the ordinal number of branch nodes, and V is the number of possible values of attribute b.

The gain ratio is defined as follows:

(8) $${Ga}_{r\left(E,b\right)}=\frac{Ga\left(E,b\right)}{U\left(b\right)},$$

(9) $$U\left(b\right)=-\sum_{v=1}^{V}\frac{\left|E^{v}\right|}{\left|E\right|}{log}_{2}\frac{\left|E^{v}\right|}{\left|E\right|},$$

The Gini index of attribute b is defined as:

(10) $$Gi\_in\left(E,b\right)=\sum_{v=1}^{V}\frac{\left|E^{v}\right|}{\left|E\right|}Gi\left(E^{v}\right)$$

(11) $$Gi\left(E\right)=1-\sum_{i=1}^{\left|o\right|}p_{i}^{2}$$

(2) ***Random Forest (RF):*** RF is an ensemble learning method employed to address classification and regression problems. It constitutes a model comprised of multiple decision trees. Ultimately, the prediction results from each decision tree are combined using methods such as averaging or voting to produce the final prediction.

The average method and the voting method are defined as Formulas (12) and (13), respectively:

(12) $$L\left(x\right)=\frac{1}{Q}\sum_{j=1}^{Q}l_{j}(x)$$

(13) $$L\left(x\right)=z_{\underset{k}{\mathrm{arg min}}\sum_{j=1}^{Q}m_{j}l_{j}^{k}(x)}$$

where Q is the number of classifiers, $l_{j}(x)$ is the output of individual learner $l_{j}$ on instance x, $m_{j}$ is the weight of individual learner $l_{j}$, and $m_{j}\geq 0$, $\sum_{j=1}^{Q}m_{j}=1$. $z_{j}$ is the number of class tags, and $l_{j}^{k}(x)$ is the output of $l_{j}$ on $z_{j}$.

(3) ***K-Nearest Neighbor (KNN):*** KNN operates on a training data set that contains labeled data points and corresponding categories or values. KNN calculates the distance between the new data point and several existing points in the training data set, and then selects the *K* neighbors that are closest. Next, the category or value of the new data point is determined either by majority vote or by averaging. Given the test sample x, if its nearest neighbor sample is t, then the probability of the nearest neighbor classifier error is the probability that x and t class labels are different, that is:

(14) $$P\left(error\right)=1-\sum_{z\in y}P\left(z|x\right)P(c|t)$$

**Figure 3:**
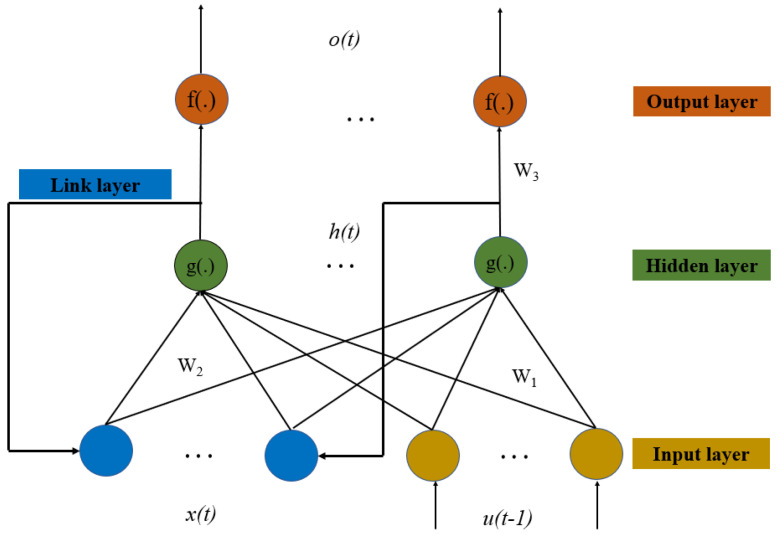
The network structure of an Elman neural network.

(4) ***Neural networks:*** Neural networks show great potential in classification and regression problem. Here, we consider several representative neural networks such as backpropagation neural networks (BPNNs) and Elman NNs. Since the BPNN deconstruction is simple and common, it will not be introduced. An Elman neural network is a typical dynamic recursive network proposed by Elman. Compared with the three-layer structure of BPNNs, the Elman neural network adds a link layer, and the output of the hidden layer at the previous time is fed back to the hidden layer as the input at the current time, which makes the network have the adaptability of time-varying characteristics, and thus increases the global stability of the network. The network structure is shown in Figure 3.

Referring to the network structure of the Elman NN in Figure 3, the relationship between input and output is given as [34]:

(15) $$\left\{\begin{array}{l} o(t)=f[w_{3}h(t)+b_{2}]\\ h(t)=g\{w_{1}[u(t-1)]+w_{2}x(t)+b_{1}\}\\ x(t)=h(t-1) \end{array}\right.$$

where *u*(*t*) is the input layer vector, *h*(*t*) is the hidden layer vector, *o*(*t*) is the output layer vector, *x*(*t*) is the link layer vector, and *t* is the moment. *w*_1_, *w*_2_, and *w*_3_ are the connection weights from the input layer to hidden layer, link layer to hidden layer, and hidden layer to output layer, respectively. *b*_1_ and *b*_2_ are thresholds of the input layer and hidden layer, respectively. *g*(*·*) is the activation function of the hidden layer, and *f*(*·*) is the activation function of the output layer. The activation functions *g*(.) and *f*(.) of the hidden layer and the output layer adopt a sigmoid function:

(16) $$f(x)=g(x)=\frac{1}{1+e^{-x}}$$

## 5. Experimental Results and Discussion

In this section, we present the process of generating channel data using both the QuaDRiGa platform and our self-developed low-orbit satellite simulation platform. We analyze how various environmental conditions affect the channel data and assess the relative significance of different channel characteristics in recognizing specific communication scenarios. Additionally, we construct a scenario recognition case and compare the recognition accuracy of different algorithms.

### 5.1. Data Generation and Analysis

QuaDRiGa was employed to generate channel data for various link types, including ground-to-ground, air-to-ground, and air-to-air. This generation takes into account three crucial environmental factors: weather conditions, noise levels, and relative mobility. Weather conditions are categorized as either rainfall or non-rainfall, noise interference is classified as low or high, and relative mobility is divided into stationary, low-speed, and high-speed conditions. Consequently, QuaDRiGa can produce channel data for a total of 36 unique scenarios. On the other hand, our self-developed low-orbit satellite communication channel simulation platform was utilized to generate channel data for link types involving space-to-space, space-to-air, and space-to-ground communication. Due to the substantial relative motion speed of low-orbit satellites, distinct numerical settings were applied to represent varying levels of relative mobility. Additionally, since space-to-space links are unaffected by weather conditions, the low-orbit satellite communication channel simulation platform generates channel data under 30 distinct scenarios. Thus, a combined total of 66 scenarios can be effectively generated to facilitate comprehensive analysis.

**Figure 4:**
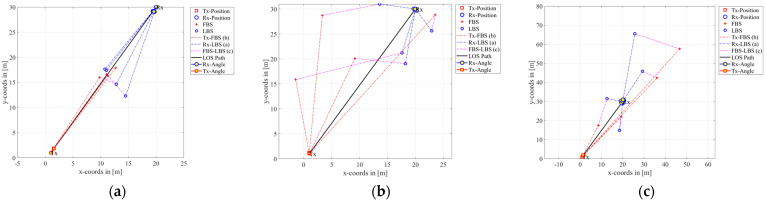
Scenario simulation using QuaDRiGa. (**a**) Tx-Position = (1, 1, 300), Rx-Position = (20, 30, 300). (**b**) Tx-Position = (1, 1, 300), Rx-Position = (20, 30, 2). (**c**) Tx-Position = (1, 1, 2), Rx-Position = (20, 30, 2).

As shown in Figure 4, QuaDRiGa was used to simulate air-to-air, air-to-ground, and ground-to-ground link communication scenarios by changing the positions of the transmitting terminal Tx and receiving terminal Rx. The figure shows the progress of a radio wave from the Tx to Rx through the first-bounce scatterer (FBS) and the last-bounce scatterer (LBS). Each line represents a multipath component with the same propagation path. The position of the scatterer is randomly generated according to the statistical law of the specific scenario.

**Figure 5:**
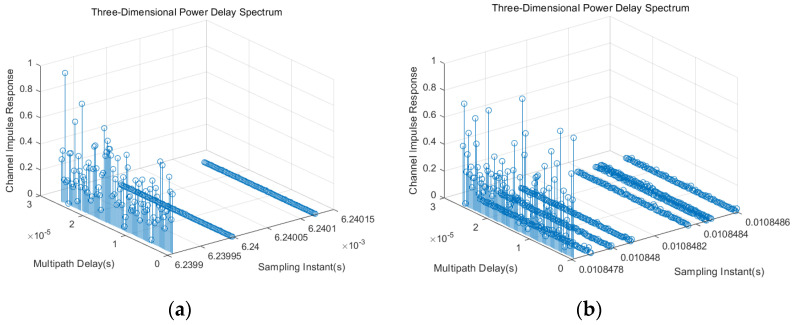
Three-dimensional power delay spectrum. (**a**) Channel impulse response in the space–ground scenario without rainfall and noise interference. (**b**) Channel impulse response in the space–air scenario with rainfall and noise interference.

The self-developed dynamic satellite channel simulation platform can obtain the large- and small-scale parameters and CIR of satellite channels. We simulated the channel impulse response of the space–ground scenario without rain and noise interference and the channel impulse response of the space–air scenario with rain and noise interference; the parameter settings of the platform are shown in Table 2, and the simulation results are shown in Figure 5. Three paths can be observed in Figure 5a, including one LoS path and two NLoS paths, and seven paths can be observed in Figure 5b, including one LoS path and six NLoS paths. Through simulation, it can be concluded that for different scenarios, the channel simulation parameters and CIR are different. Therefore, the proposed satellite simulation platform supports the modeling of multiple different scenarios.

**Figure 6:**
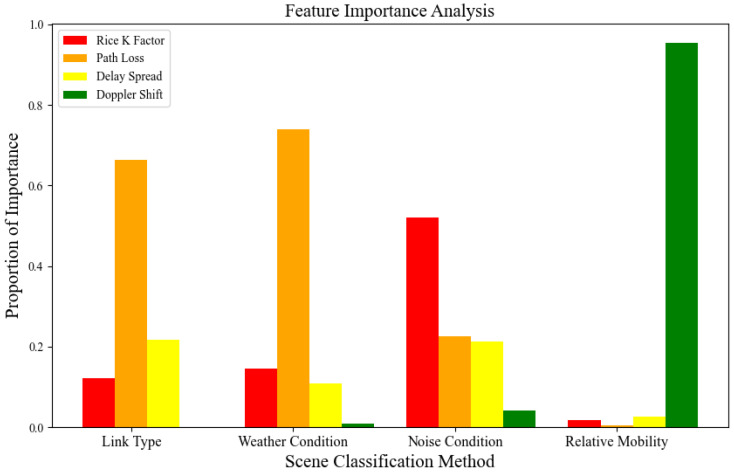
The importance analysis of channel features for different classification methods.

After the channel features are extracted from the original channel data, it is necessary to analyze the importance of the channel features. For different scenario recognition problems, the most relevant features are usually selected for training. We used random forests to assess the importance of features (results are shown in Figure 6) which lays the foundation for building intelligent recognition models in the next section.

### 5.2. Analysis and Comparison of Scenario Recognition Models Based on Different Algorithms

First, we built the scenario recognition model based on the random forest algorithm, which can be used to identify link types (space-to-space, space-to-air, space-to-ground, air-to-air, air-to-ground, and ground-to-ground), weather conditions (whether it is raining or not), noise conditions (with or without noise), and relative mobility conditions (static, low speed, and high speed). Relevant channel characteristics are selected as the input of the model according to Figure 6. As there are numerical differences in Rice *K* factor, path loss, delay expansion, and Doppler shift, they need to be normalized, and the model output is the corresponding label, with a value range of [1,2,3,…n]. Confusion matrices are a common way to evaluate the prediction effect of classification models. It is represented by an *n* × *n* matrix, where *n* represents the number of categories. The rows of the confusion matrix represent the true categories, and the columns represent the predicted categories. Each element *C_ij_* in the matrix represents the number of samples that are actually class *i* but are predicted to be class *j*. In order to further understand the results of data prediction and improve the prediction method, a confusion matrix is drawn to observe the performance of the recognition model.

**Figure 7:**
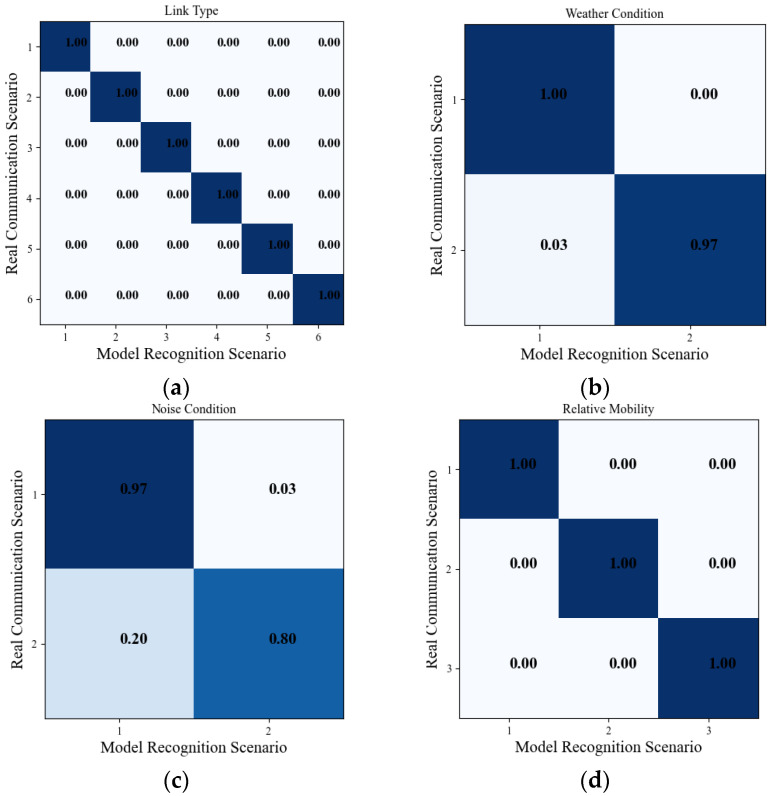
Confusion matrix for different scenarios. (**a**) Link types; (**b**) weather conditions; (**c**) noise conditions; (**d**) relative mobility conditions.

We used a confusion matrix to give the recognition results of the random forest-based recognition model for link type, weather condition, noise condition, and relative mobility, as shown in Figure 7. As can be seen from the figure, the prediction accuracy of most link type labels was above 90% and the prediction rate was above 70%; the prediction accuracy of weather condition labels was above 90%; the prediction accuracy of noise condition labels was above 90%; and the prediction accuracy of relative mobility labels was above 98%. These prediction results are within the allowable error range. Therefore, the validity of the model was proven and the importance of channel feature importance evaluation is reflected.

**Figure 8:**
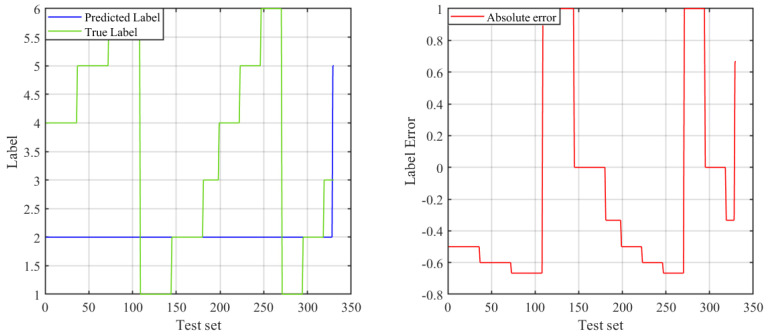
Prediction of link types based on Elman neural network.

**Figure 9:**
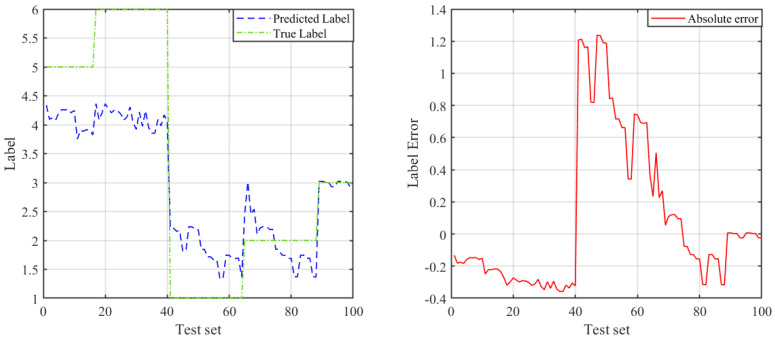
Prediction of link types based on BAS–Elman neural network.

Then, we use an Elman neural network to build model to predict the link types (numbers 1–6 represent the six scenarios of “space-to-ground”, ”space-to-air”, “space-to-space”, ”air-to-air”, “air-to-ground”, and “ground-to-ground”, respectively). The recognition results are shown in Figure 8, and it can be seen that the prediction effect is not satisfactory. Then, the beetle antenna search (BAS) algorithm [26] was used to optimize the network weight of the Elman neural network to improve its learning performance. The prediction result of Elman after BAS optimization is shown in Figure 9. Comparing the two figures, it can be seen that the prediction accuracy of Elman after BAS optimization has improved.

We also built recognition models based on KNN and BP neural networks, so we summarized the accuracy of the methods used in this paper for different classification problems into Table 3. The classification problems considered are given in the first row, and the machine learning methods adopted are given in the first column. As can be seen from the table, the recognition accuracy of the random forest algorithm is at a high level under various classification problems, and the recognition stability of KNN algorithm is weak, while that of BP algorithm is strong. In addition, the recognition accuracy of the Elman algorithm optimized by BAS is significantly improved.

## 6. Conclusions and Future Work

This paper proposes a simulation framework for SAGIN communication propagation channel research, which integrates the existing QuaDRiGa channel simulation platform and a self-developed low-orbit satellite simulation platform. According to the process of the framework, the channel data in various scenarios are simulated first, and the channel characteristics are extracted by statistical methods and the importance analysis on channel characteristics is carried out. Then, based on different methods, the recognition models are constructed to classify link types and environmental conditions such as weather condition and noise interference, and the comparative analysis is carried out. The experimental results verified the rationality of our proposed framework, which can be used to assist researchers in studying communication propagation channels.

We believe our proposal is a useful asset for SAGIN propagation channel research, which is an interesting direction for future research as it compensates for difficulties in real-world testing. There are several directions for future work: one is to extend our simulation framework to manage smart cities in areas such as network performance analysis and optimization; the second is the optimization of simulation fidelity, such as the accurate modeling of time-varying channels and communication nodes under environmental interference; and finally, the integration and ease-of-use of the simulation framework will be further improved to provide simulation services for complex tasks.

## Figures and Tables

**Table 1:** List of important notations for channel characteristics.

Notations	Definitions
*PL*	Path loss
α	Path loss factor
$P\left(\psi \right)$	Shadowing
ψ	Transmit–receive power ratio
K(dB)	*K* factor
$\bar{\tau }$	Average time delay
$a_{k},\;\tau_{k},\;P(\tau_{k})$	Amplitude, delay, and energy of the *k*th path
$f_{d}$	Doppler shift
*AS*	Angular spread
$\varphi_{k}$	Angular delay of the *k*th path

**Table 2:** Parameter settings of satellite dynamic channel simulation platform.

Parameter	Value
Communication frequency	3.6192 GHz
Number of originating antennas	1
Number of receiving antennas	1
Rainfall rate	10 mm/h
Longitude of the ground station	114°
Latitude of the ground station	41°
Semi-major axis	7371 km
Eccentricity	0
Orbital inclination	65°
The perigee angle	0°
Right ascension of ascending node	300°
Flat near point angle	0°

**Table 3:** Overall accuracy using different classifiers and features.

	Classifier				
Classification Mode	KNN	Random Forest	BP	Elman	Bas_Elman
Link type	67.68%	89.39%	91.60%	70.65%	78.63%
Weather condition	96.97%	96.39%	94.08%	78.03%	83.57%
Noise condition	75.76%	92.77%	94.74%	74.16%	79.05%
Relative mobility	99.79%	99.39%	92.94%	80.01%	79.93%

## Data Availability

The data that support the findings of this study are available from the corresponding author, [Men, W.], upon reasonable request.
